# Biogeographic and diversification patterns of Neotropical Troidini butterflies (Papilionidae) support a museum model of diversity dynamics for Amazonia

**DOI:** 10.1186/1471-2148-12-82

**Published:** 2012-06-12

**Authors:** Fabien L Condamine, Karina L Silva-Brandão, Gael J Kergoat, Felix AH Sperling

**Affiliations:** 1 INRA, UMR Centre de Biologie pour la Gestion des Populations, CBGP, (INRA/IRD/CIRAD/Montpellier SupAgro), Campus International de Baillarguet, CS30016, 34988, Montferrier-sur-Lez, France; 2 CNRS, UMR 7641 Centre de Mathématiques Appliquées (École Polytechnique), Route de Saclay, 91128, Palaiseau, France; 3 Departamento de Entomologia e Acarologia, Escola Superior de Agricultura “Luiz de Queiroz”, Universidade de São Paulo, Av. Padua Dias 11, Piracicaba, SP, Brazil, 13418-900; 4 Department of Biological Sciences, University of Alberta, Edmonton, AB, Canada, T6G 2E9

**Keywords:** Amazon rainforest, Andean uplift, Biogeography, Diversification, GAARlandia connection, Swallowtail butterflies

## Abstract

**Background:**

The temporal and geographical diversification of Neotropical insects remains poorly understood because of the complex changes in geological and climatic conditions that occurred during the Cenozoic. To better understand extant patterns in Neotropical biodiversity, we investigated the evolutionary history of three Neotropical swallowtail Troidini genera (Papilionidae). First, DNA-based species delimitation analyses were conducted to assess species boundaries within Neotropical Troidini using an enlarged fragment of the standard barcode gene. Molecularly delineated species were then used to infer a time-calibrated species-level phylogeny based on a three-gene dataset and Bayesian dating analyses. The corresponding chronogram was used to explore their temporal and geographical diversification through distinct likelihood-based methods.

**Results:**

The phylogeny for Neotropical Troidini was well resolved and strongly supported. Molecular dating and biogeographic analyses indicate that the extant lineages of Neotropical Troidini have a late Eocene (33–42 Ma) origin in North America. Two independent lineages (*Battus* and *Euryades* + *Parides*) reached South America via the GAARlandia temporary connection, and later became extinct in North America. They only began substantive diversification during the early Miocene in Amazonia. Macroevolutionary analysis supports the “museum model” of diversification, rather than Pleistocene refugia, as the best explanation for the diversification of these lineages.

**Conclusions:**

This study demonstrates that: (*i*) current Neotropical biodiversity may have originated *ex situ*; (*ii*) the GAARlandia bridge was important in facilitating invasions of South America; (*iii*) colonization of Amazonia initiated the crown diversification of these swallowtails; and (*iv*) Amazonia is not only a species-rich region but also acted as a sanctuary for the dynamics of this diversity. In particular, Amazonia probably allowed the persistence of old lineages and contributed to the steady accumulation of diversity over time with constant net diversification rates, a result that contrasts with previous studies on other South American butterflies.

## Background

The Neotropical ecozone, including its Central and South American regions, is arguably the most species-rich terrestrial biogeographic region on Earth [1]. The primary hypothesis, considered the most likely explanation for understanding the origin and diversification of Neotropical biodiversity, suggests that this diversity arose by *in situ* speciation [2-4]. However, several recent phylogenetic studies have revealed that a substantial part of the extant diversity of this region can be accounted for by *ex situ* origins and dispersals of taxa into the Neotropics (e.g., [5-8]). These lineages subsequently diversified and became major components of communities within the Neotropical biota, both in term of species richness and ecological dominance [9]. The mode of diversification of this extraordinary high species diversity is often attributed to two competing hypothetic models. The ‘museum’ model postulates that gradual accumulation and/or preservation of species over time via constant speciation rates and/or low extinction rates have shaped the present diversity [10,11]. In contrast, the ‘evolutionary cradles’ model predicts that the extant diversity is the result of recent and rapid accumulation of species via high speciation rates [4,12]. Although ‘evolutionary cradles’ and ‘museum’ models are presented as alternative hypotheses explaining the diversification of many groups, both models can account for the diversification of Neotropical taxa [13], rendering the understanding of Neotropical diversification more intricate than expected [9]. Although the age and geographic origin of many Neotropical groups is well known (e.g., [6-8]), less is known about their diversification patterns [14]. Similarly, the factors that have shaped this high species richness through time have been scarcely investigated in a large-scale and temporal framework. This is especially true for the processes that underlie the assembly and evolution of Amazonian biodiversity [15-17].

Among the potential factors influencing diversification patterns, geological history (tectonic movements and mountain orogenesis) has had profound consequences for the origin and evolution of Neotropical biodiversity through the rise and fall of biogeographic barriers [6,18]. For instance the Andean uplift affected regional climate, which in turn dramatically changed the Amazonian landscape by reconfiguring drainage systems [15]. Climate change is often postulated as a major driver of present-day biodiversity pattern, thus favouring the ‘evolutionary cradles’ model, but investigating its effects through time remains challenging [16,19-21]. In addition, differences in clade age may underlie variation in species richness among lineages (i.e., ‘time-for-speciation hypothesis’; [22,23]), while speciation rates may be intrinsically higher (or extinction rates lower) in clades during the periods that they occupy tropical versus temperate regions, even if the clades are the same age (i.e., ‘diversification rate hypothesis’; [24]). Finally, biotic effects such as host plant interactions can contribute to the geographic diversification of a group [25,26]. These distinct factors are potentially linked and interwoven, and it is difficult to disentangle and quantify their respective contribution to the establishment of Neotropical diversity [17]. As advocated by several authors, solving this puzzle requires the combination of multiple research fields such as biogeography, climatology, ecology, evolutionary biology, paleogeography, and palaeontology (e.g., [13,17]).

In this study, we investigate the diversification patterns of three Neotropical swallowtail genera from the tribe Troidini (Lepidoptera, Papilionidae). This tribe comprises some of the most spectacular butterflies on Earth and represents one of the best-studied swallowtail groups (e.g., [27-29]). Troidini are predominantly tropical and include about 130 species divided into 12 genera, with most species encountered in the lowland rainforests of the Neotropical and Indo-Australian regions [30]. We focused on the New World Troidini, which include three genera: *Battus* (10 to 12 species), *Euryades* (2 species), and *Parides* (up to 35 species) [27,29]. These three genera do not form a monophyletic clade [28,31]. The genus *Battus*, recovered as sister to the remaining Troidini, is distributed from North America to southern South America, with its species richness increasing towards the equator [28]. Exclusively Neotropical, the genus *Parides* is the most diversified genus of Troidini [29]. Many subspecies or varieties have been described, possibly constituting species of their own. The genus *Euryades*, sister to *Parides*, has a peculiar distribution confined to the Cerrado region (south-eastern South America). Interestingly Troidini are frequently cited as classic examples of coevolution with their host plants *Aristolochia*[32,33], and are thus commonly called pipevine swallowtails. The larvae of Troidini feed exclusively on *Aristolochia* species, and sequester the major secondary metabolites of these plants [33]. Although this association partially agrees with the premises of the coevolution hypothesis [32], Silva- Brandão *et al.*[34] demonstrated that there is no strict codiversification pattern between the insects and plants, suggesting that their diversification was probably not driven by their host plant history. This hypothesis was partially confirmed by a study of Fordyce [35] that found no evidence of diversification rate shifts within Aristolochiaceae feeders throughout their history.

We have previously demonstrated that the extant diversity of troidines from the Western Hemisphere had a boreotropical origin followed by southward dispersal of taxa into the Neotropical region [36]. We thus focus here on exploring the tempo and mode of diversification of Troidini within the Neotropics. To better understand this diversity pattern, a species-level and molecularly dated phylogenetic hypothesis is necessary to assess and quantify the possible factors and mechanisms that have shaped their present Neotropical biodiversity through time. Until now, few studies have focused on the internal relationships of the three genera (but see [27,28]), and even fewer have tried to assess their divergence times. Using a molecular phylogenetic framework for the New World Troidini of the genera *BattusEuryades* and especially *Parides*, this study aims to: (*i*) use a DNA-based delimitation method to clarify the species status of several Troidini species to provide better estimates of species richness; (*ii*) infer their historical biogeographic history using a Bayesian relaxed-clock method and model-based geographic analyses; and (*iii*) investigate their diversification pattern to link possible changes in diversification rates with proximate mechanisms in past abiotic and/or biotic events.

## Methods

### Taxon sampling and Molecular data

Overall, our taxon sampling includes five *Battus* out of 11 described species, the two described *Euryades* species, and 21 *Parides* species out of 34, representing all species groups (*sensu*[29]). The taxonomic coverage encompasses about 60% of all Neotropical Troidini species (Additional file 1 Dataset S1). Outgroup selection is a crucial step in phylogenetics, especially in estimates of divergence times [37]. Based on the most comprehensive and recent phylogeny of swallowtail butterflies [36], three outgroups were chosen. These species (*Graphium agamemnonPapilio machaon*, and *Teinopalpus imperialis*) were included as they represent sister tribes to the Troidini (Leptocircini, Papilionini and Teinopalpini respectively; [31]). Sampled taxa and GenBank accession numbers for all materials are given in Additional file 1 Dataset S1.

All molecular data on Neotropical Troidini were retrieved from previously published studies on the GenBank database [28,30,31,34,38,39]. About 2.3 kilobases of two mitochondrial genes were used, namely cytochrome oxidase I (COI) and cytochrome oxidase II (COII), and about 1.2 kilobases of the nuclear protein-coding gene elongation factor 1 alpha (EF-1α). The phylogenetic utility of these genes has been widely demonstrated for Papilionidae (e.g., [36]), and especially for Troidini [28,30]. In summary, sequence for 165 specimens for COI, 71 specimens for COII, and 50 specimens for EF-1α have been retrieved (including outgroups). These sequences were aligned using ClustalX 2.0 with the default settings [40]. The reading frame of coding genes was further checked under Mesquite 2.75 (available at: http://www.mesquiteproject.org).

### DNA-barcode marker and species delimitation procedure

The aim of tree-based DNA species delimitation is to classify observed branching time intervals defined by nodes in a clock-constrained phylogeny as either being the result of inter-specific (diversification) or intraspecific (coalescent) processes of lineage branching [41]. In our study, this approach was implemented to provide an objective assessment of the number of genetic lineages that correspond to species, the latter entities being crucial to all biogeographic and diversification analyses [42]. Although the results of DNA-based genetic lineage delimitation can also be used to clarify and test the species taxonomy of the group, we have chosen to refer to these entities in a conservative manner by retaining all original species names associated via morphology with the specimens. Numbers at the end of the species name distinguish distinct molecular entities within a morphologically defined species.

The General Mixed Yule Coalescent model (GMYC) was employed to perform analyses of species delimitation [41]. This method is implemented in R software and uses the *splits* (available at http://r-forge.r-project.org/projects/splits/) and the *ape*[43] packages. This approach usually relies on a single threshold to delimit nodes defining the most recent common ancestor of species.

Phylogenetic analyses were performed on the COI mitochondrial gene, well known as the standard barcode region for animals [44]. Here we used the entire COI gene (1,527 bp) rather than the standard 648 bp fragment [44]. We did not perform these phylogenetic analyses with the whole molecular dataset because there are missing data for the other genes (i.e., only 50 specimens for EF-1α versus 165 specimens for COI) that can alter some parameters such as branch length [41]. However, a phylogeny using only the EF-1α gene was also constructed as an independent genomic test of the COI-defined molecular lineages. This provides a practical and conceptual link between molecular entities defined solely by single-locus barcodes and species as population units defined by maintenance of their genomic integrity, equivalent to reproductive isolation.

Bayesian phylogenetic inference was performed to recover relationships (see below for details on settings for phylogenetic reconstructions). From the COI-phylogeny, the program PATHd8 [45] was used to transform the tree (with branch lengths scaled as evolutionary rate) into an ultrametric tree (with branch lengths proportional to time) using the mean path length algorithm. When applying the GMYC model on the ultrametric tree [41], a transition in branching rates may be identified as a sudden increase in slope of the plot. We compared the likelihood of the null model (assuming a single branching process for the tree) to the GMYC model (assuming significant changes in branching time). The threshold model has five parameters (λ1, speciation rate before the threshold; λ2, speciation rate after the threshold; p1, scaling parameter before the threshold; p2, scaling parameter after the threshold; and T, threshold time), whereas the null model has two (λ1 and p1); hence, there are three degrees of freedom (d.f.) for the comparison.

The delimitation of molecular species was then used to build a ‘reduced dataset’. This new dataset comprised a single specimen for each putative species cluster (molecular entity) obtained by reconstructing the consensus of all the sequences recovered in the same species cluster. The consensus sequences were inferred by using Mesquite with default settings. Then we combined COII and EF-1α sequences with COI-consensus sequences. This ‘reduced dataset’ was further used to reconstruct a species-level phylogeny to estimate the divergence times, geographic range evolution and diversification rates of Neotropical troidine species.

### Species-level phylogenetic analyses

Phylogenetic analyses were performed with probabilistic methods (maximum likelihood, (ML) and Bayesian inference (BI)). The molecular dataset was combined and partitioned into six partitioning strategies (PS): (*i*) a single partition encompassing all genes (PS1), (*ii*) two partitions (one partition for the mitochondrial genes and one partition for the nuclear gene; PS2), (*iii*) three partitions (one partition per gene; PS3); (*iv*) three partitions (one partition per codon position; PS4); (*v*) six partitions (one partition per codon position for the mitochondrial coding genes, one partition per codon position for the nuclear coding gene; PS5); and (*vi*) nine partitions (one partition per codon position for each protein-coding gene; PS6). For each partition, the substitution model of sequence evolution was selected using jModelTest 0.1 [46] with the Bayesian information criterion (BIC) [47]. The General Time Reversible (GTR) + I + Γ model was recovered for all partitions, except those using codon positions (PS4-6) where the second position gave F81 + I + Γ and the third position consistently gave GTR + Γ. The tRNA-leucine sequence between COI and COII was not coded with a substitution model but instead treated as non-coding.

Maximum likelihood analyses were carried out using PhyML 3.0 [48] to obtain fast and accurate phylogenetic results. All the analyses were parameterized with the best-fit substitution model, the fastest algorithm to perform Nearest Neighbor Interchanges and also the algorithm Subtree Pruning and Regrafting [48], a BIONJ starting tree, 1,000 bootstrap replicates, and the remaining parameters set to default settings. Independent runs were carried out to check congruence in likelihood scores [49]. Bootstrap values (BV) ≥ 70% were considered as moderate support whereas BV ≥ 90% indicated strong support for a node [49].

Bayesian inference analyses were performed with MrBayes 3.1.2 [50] with the following settings: (*i*) two independent runs with eight Markov Chains Monte Carlo (MCMC, one cold and seven incrementally heated), (*ii*) 10.10^6^ generations for the complete dataset, (*iii*) the trees were sampled every 100th cycle and each MCMC started from a random tree. To generate the 50% majority rule consensus tree, a conservative burn-in of 25% was applied after checking the log-likelihood scores and the split-frequencies of the runs, and all sampled trees prior to reaching these generations were discarded. Posterior Probabilities (PP) estimated the node supports, and PP ≥ 0.95 were usually considered as strong support [49]. The potential scale reduction factors (PSRF) were checked after the end of each analysis and should approach 1 as runs converge [49].

Selection of the best-fit PS (with the best-fit substitution model) was performed using Bayes Factors (BF) for BI [47,51]. Estimates of harmonic mean (HM) of the likelihood values were obtained with the *sump* command in MrBayes, and were used to approximate the BF between two substitution models or partitioning strategies. BF values > 10 were considered to significantly favour one model over another [47,51]. Hypothesis tests (e.g., monophyletic constraints) were conducted by comparing likelihood scores with SH-tests [52] in ML using 1,000 RELL bootstrap replicates, and with BF approximated by harmonic means in BI.

### Bayesian estimates of divergence times

The applicability of a molecular clock was assessed for each node using PATHd8 [45]. Because the hypothesis of a molecular clock was not statistically supported for our dataset, a Bayesian relaxed-clock (BRC) approach was used to take into account rate variation across lineages [53]. It uses MCMC procedures to approximate the posterior distribution of rates and divergence times and simultaneously infer their credibility intervals. The BRC analyses were carried out with BEAST 1.6.2 [54]. We used the standard log-normal model to account for changes in evolution rates, the latter being uncorrelated in BEAST. The xml-file for BEAST analyses was created using the BEAUti interface (included in the BEAST package) with the following non-default settings and priors: the *Site Model* was set to the same nucleotide substitution model evolution, with partitioning (1 + 2) and 3 for all partitions, the *Clock Model* was set to a relaxed clock with uncorrelated rates, the *Tree Model* was set to a Yule process of speciation, and the *MCMC parameters* were fixed to 5.10^7^ generations with sampling every 1,000 generations and the first 25% discarded as burn-in. The starting tree was enforced with the best MrBayes topology defined by Bayes factors. The remaining parameters were left unchanged. In BEAST analyses, only a single MCMC explores the data; thus several independent analyses were performed to check congruence of results and likelihood scores. The output files were combined and checked in LogCombiner 1.6.2 (included in the BEAST package), and also checked in Tracer 1.5 [54]. Under Tracer, an effective sample size (ESS) superior or equal to 500 for all parameters was used to compare the analyses. Chronograms from BEAST are maximum clade credibility trees with the 95% highest posterior density (95% HPD) rescaled to match posterior median estimates, compiled from posterior trees using TreeAnnotator 1.6.2 (included in the BEAST package).

The BRC approach also allows using flexible techniques for incorporating calibrations leading to discussion about approaches to calibrating estimates of divergence times [53]. The selection of calibrations is an important issue because divergence time estimates are taken into account by the biogeographic analyses used in this study [55]. For swallowtails, the fossil record is scarce and the known unambiguous swallowtail fossils do not belong to the Neotropical Troidini genera. Therefore, secondary calibrations were chosen to calibrate specific nodes. Calibrations were retrieved from a previous study that estimated the divergence times of the Papilionidae using the most comprehensive taxon sampling and molecular dataset [36]. As a similar phylogenetic pattern was recovered for Troidini in this study, dates were constrained for each corresponding node, using the 95% HPD values. Seven calibration points were set to uniform distribution following the recommendation of Ho and Phillips [37]. The prior distribution for the root time (*Root Height*) was constrained to be within 40.61–57.39 Ma (corresponding to the 95% HPD for the age of Papilioninae), the node between Troidini and Papilionini was set to 37.23–53.05 Ma, and the node between Papilionini and Teinopalpini was set to 33.16–48.64 Ma. Finally Troidini was constrained to 32.45–46.92 Ma, *Battus* was constrained to be within 17.22–29.29 Ma, *Euryades* was set to 7.21–16.9 Ma, and *Parides* constrained to 18.56–28.45 Ma [36].

### Historical biogeography

**Figure 1:**
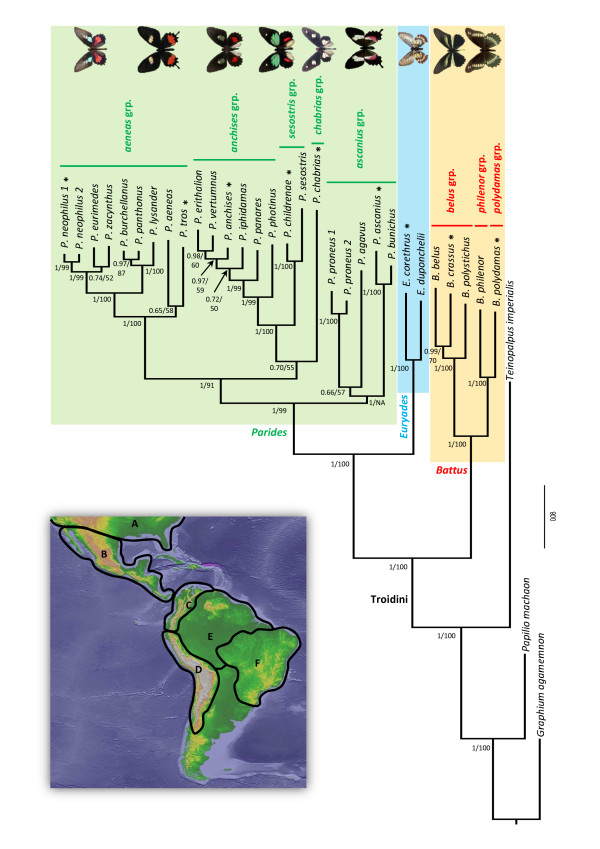
**Phylogeny of Neotropical Troidini obtained with the best-fit partitioning strategy (six partitions) under Bayesian inference.** Posterior probabilities (PP) and maximum likelihood bootstrap values (BV) are shown by nodes. NA indicates that ML analyses did not recover this branching. Coloured tinted boxes highlight the three genera, and the species groups are also indicated within *Battus* and *Parides*. For illustration purposes, images of some species are shown beside the tree for species with asterisks (*). The geographic map shows areas used in biogeographic analyses (see Materials and Methods for details).

Reconstruction of ancestral areas was inferred using the Dispersal–Extinction–Cladogenesis (DEC) model of range evolution, as implemented in the software Lagrange [55]. The distribution of New World Troidini extends from the Nearctic to the Neotropics, which were subdivided into smaller biogeographic units. For that purpose, we followed several studies [6,15,16] and used several lines of evidence to define the areas, such as paleogeography (e.g., [56]) or biodiversity hotspots [1]. The model comprised six component areas, namely (A) Nearctic (North America up to Mexico); (B) Mexican region (i.e., Central America, including Northern Mexico up to the Isthmus of Panama); (C) Northern South America (including the Chocó and Páramo regions, the Northern Andes and the Guiana shield); (D) Central Andes (all along the South America); (E) Amazonian forest; and (F) Brazilian shield (South-Eastern South America, including the Cerrado region and Brazilian Atlantic forest) (see inset in Figure 1). Species ranges were finally coded by presence–absence data, excluding marginal distributions or known human introductions [6,57].

Among the theoretically possible geographic ranges, some were excluded from consideration based on the biological implausibility of their spatial configurations (e.g., wide disjunction between A and F). We thus defined a biogeographic model by taking into account the geological history of the region. Following the principles described in Ree and Smith [55], temporal constraints on rates of dispersal were designed between areas based on paleogeographic reconstructions of area position through time (e.g., [56]). Constraints were implemented as a series of five time slices:

(1) Origin of Troidini up to 32 Ma. Amazonian forest extended north to the Caribbean coast of South America where a diverse rain forest existed [15]. Andes orogenesis had just begun, and the Pozo embayment separated the northern and central Andes. In the North, GAARlandia (Greater Antilles and Aves Ridge land bridge; [58]) connected the Nearctic and the Neotropics [15].

(2) 32 to 23 Ma. This period corresponded to the presence of a sub-Andean river system where South America was a lowland river-dominated landscape [15]. GAARlandia disappeared [58], as well as the Pozo embayment, leaving space for the western Andean portal [6]. This was also a period of intensified Andean uplift.

(3) 23 to 10 Ma. This time was characterized by the intensification of the Andean uplift and subsequent changes in Amazonian landscape with the formation of the Pebas system separating western and eastern South America [15]. This period is noteworthy for numerous evolutionary radiations recorded in central Amazonia [5, 6, 14, 59].

(4) 10 to 7 Ma. The Amazon River originated during this time. As a result of the Andean uplift, the drainage system changed and fragmented the Amazonian forest with the formation of the Acre system [15].

(5) 7 Ma to present. The Amazon River is fully established. This period corresponds to the formation of the Isthmus of Panama connecting Central and South America [15, 60].

For each time slice, a matrix of scaling factors (between zero and 1) was constructed for setting dispersal rate between areas according to their geographic position, interpreting greater distances and/or the extent of geographic barriers (e.g., sea straits, mountain chains) as being inversely proportional to the expected rate. The paleogeographical model used in this study analysis, with five time slices reflecting the probability of area connectivity through time, is given in Additional file 2 Dataset S2.

The DEC method allows performance of local optimization for the root in a statistical framework [55]. Concerning the Neotropical Troidini root, we chose to constrain the origin of the group in the Nearctic (A) because Condamine *et al.*[36] demonstrated that the New World Troidini originated in the Nearctic and further dispersed to Neotropics, where the highest current diversity is located [29]. Thus, no specific tests were performed in which the root was constrained to be another area. The numbers of areas (*maxareas*) optimised for a node have been constrained to be no more than two areas, but we have performed analyses with three maximum areas per node and we obtained similar results. Overall, an area (or combination of areas) was considered to be significant if a difference of 2-log likelihood units is recovered between other areas [55].

### Temporal shifts in diversification

To investigate the tempo and mode of diversification within the Neotropical Troidini, we followed a step-by-step procedure under the program R with the *ape*[43], *geiger*[61] and *laser*[62] packages. First, using the time-calibrated phylogeny, lineages-through-time (LTT) plots were reconstructed to graphically visualize the pattern of diversification rates through time. Then, the overall diversification rate was estimated under a simple birth-death model [63], with net diversification rates resulting from differences between speciation and extinction rates. All these analyses were performed using three distinct extinction rates (ϵ = 0/0.5/0.9) and also took into account the taxa that were not sampled.

To test whether the evolution of Neotropical Troidini follows a ‘museum’ or an ‘evolutionary cradle’ model of diversification, two methods were used: γ-statistics [64] and a likelihood-based method [42,65]. The ‘museum model’ hypothesis, which predicts that speciation and extinction rates are constant through time, is considered as the null hypothesis under the γ-statistics fitting a pure birth (Yule) process (constant-rates test; [64]). In case of rejection, the ‘evolutionary cradle’ model is then favoured. This test was computed with the chronogram of Troidini (outgroups removed) and was also calculated by taking into account the missing taxa, as our taxon sampling was not exhaustive. For this, we employed Monte Carlo simulations (MCCR-test) using the number of known species (47) plus the putative species revealed by the GMYC analyses, and the number missing (17), the observed γ-statistics and fixing the number of replicates to 10,000 as advised by Pybus and Harvey [64].

The likelihood-based method used in this study compares several diversification models with a constant diversification rate (RC-models) with variable diversification rate models (RV-models) [42]. Constant rate and variable rate models were compared by AIC and likelihood ratio tests [42] computed under the program R. The lowest AIC indicates the model that best approximates the data. Difference in AIC scores between the best RC and RV models is calculated as: ΔAIC_RC_ = AIC_RC_ – AIC_RV_[62]. Following Rabosky [42], the RC model can only be rejected with confidence when ΔAIC_RC_ approaches 4.0 for small phylogenies (n = 30), and 5.5 for large phylogenies (n = 100).

Finally, the hypothesis of rate shifts occurring at major geological and climatic events during the evolutionary history of Neotropical Troidini was also assessed by likelihood methods. The approach compares models with a varying diversification rate on specific time intervals with a given time shift [42]. For instance, if a major climate change occurred at a given time, it is possible to compare the diversification rates before and after this event, and to assess their significance under likelihood analyses [26].

## Results and discussion

**Figure 2:**
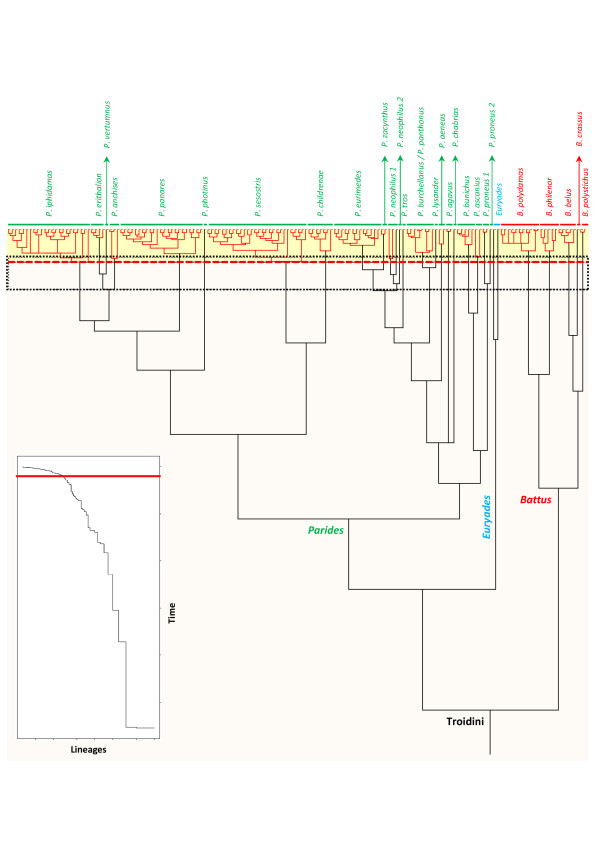
**COI-based species delimitation analysis using the method of Pons *et al.* (2006).** The Neotropical Troidini ultrametric tree obtained with PATHd8 shows clusters of specimens recognized as putative species. Genetic lineages recognized as putative species are highlighted in red and separated by longer black branches. The top corner graph shows the lineages-through-time plot based on the ultrametric tree. The sudden increase in branching rate, indicated by a red line, corresponds to the shift from interspecific (orange rectangle) to intraspecific (yellow rectangle) lineage branching. The vertical bars at the end of the branches group all sequences within each significant cluster, labelled by a provisional species name.

The evolutionary history of New World Troidini was reconstructed with multigene data (3,374 bases) and phylogenetic analyses (Figures 1 and 2). Phylogenetic analyses (ML and BI) recovered a similar, well-resolved and supported phylogenetic framework because 71% of BV nodes were ≥ 70% (ln *L* = −20,346) and 84% of PP nodes were ≥ 0.95 (Figure 1). For Bayesian analyses, Bayes factors indicate that the best-fit PS is PS5 (six partitions, HM _PS1_ = −20,355.30; HM _PS2_ = −20,009.75; HM _PS3_ = −219,989.13; HM _PS4_ = −20,120.11; HM _PS5_ = −18,716.35; HM _PS6_ = −19,189.94). All other PSs yield a similar topology and all PSRF approached 1.0 showing that the BI runs converged correctly. DNA-based species delimitation supported assembly of a reduced dataset comprising a single ‘putative species’ per molecular cluster (Figure 2; see below for details on species delimitation analyses).

According to the phylogenetic results, we recovered each genus as a monophyletic group with strong support in BV and PP (>70 and >0.95 respectively; Figure 1). A unique ambiguity is found within the genus *Parides* where topologies with either four or three clades are recovered in ML and BI respectively. We then constrained the tree to fit either a three or four clade topology in ML and BI respectively. Their likelihood scores were further compared with SH-test [52] and BF [47,51]. Both analyses confirmed that the four-clade topologies are not significantly better supported than the three-clade topologies because the likelihood in the ML analysis is not statistically different (ln *L*_Constrained_ = −20,299; *p* > 0.05 for SH-test) and the harmonic mean has increased in BI (HM _Constrained_ = −18,733.60; BF < 10). Because Silva-Brandão *et al.*[28] and Condamine *et al.*[36] also recovered the three-clade topology, it was preferentially used to investigate the tempo and mode of diversification of Troidini.

### Through GAARlandia to Amazonia: evolutionary radiation of Neotropical Troidini

**Figure 3:**
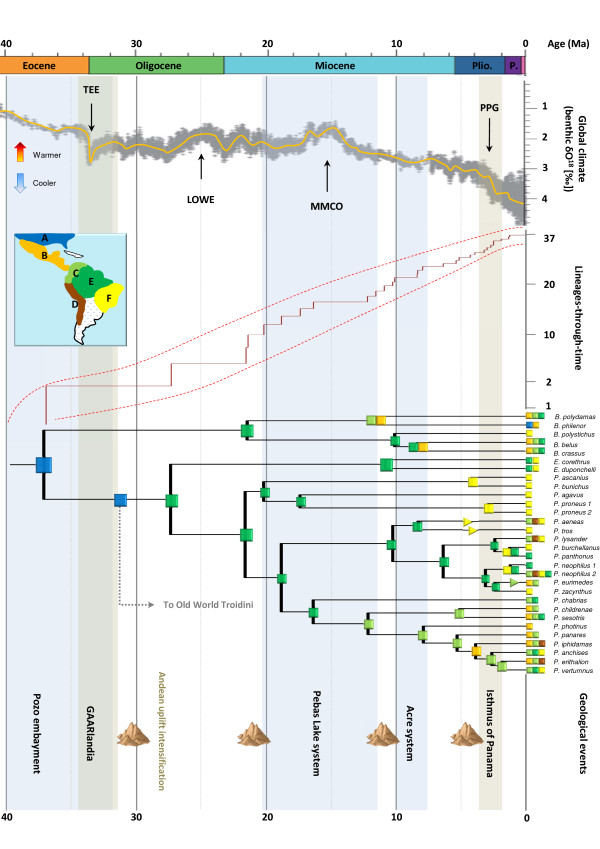
**Origin and diversification pattern of Neotropical Troidini.** The uppermost panel shows the evolution of paleoclimate as estimated by proxy Benthic δO^18^[20]. Major climate events are indicated. The lineages-through-time plot is placed next, with the 95% confidence interval indicated by a dotted red line. Most likely biogeographic reconstructions (inferred by the DEC model) are shown on the median-age BEAST chronogram of Neotropical Troidini. Major geological events are also indicated to show congruence between speciation and possible factors of diversification.

Our molecular dating analyses suggest that Troidini evolved in the late Eocene/early Oligocene boundary around 37 Ma with a 95% HPD between 32.5–42.2 Ma (Figure 3; maximum clade credibility tree with median ages from the Bayesian uncorrelated lognormal method is provided in Additional file 3Dataset S3). Most importantly, our dating and biogeographic analyses elucidate the colonization of South America by Troidini, indicating that these swallowtails have independently colonized South America twice from the Nearctic region because *Battus* and *Euryades* + *Parides* are not monophyletic within the tribe (see [28,30,36]). The ancestor of *Euryades* and *Parides* and that of *Battus* diversified in South America around 27 Ma (95% HPD 23–32.4 Ma) and 21 Ma (95% HPD 17.2-26.5 Ma) respectively.

**Figure 4:**
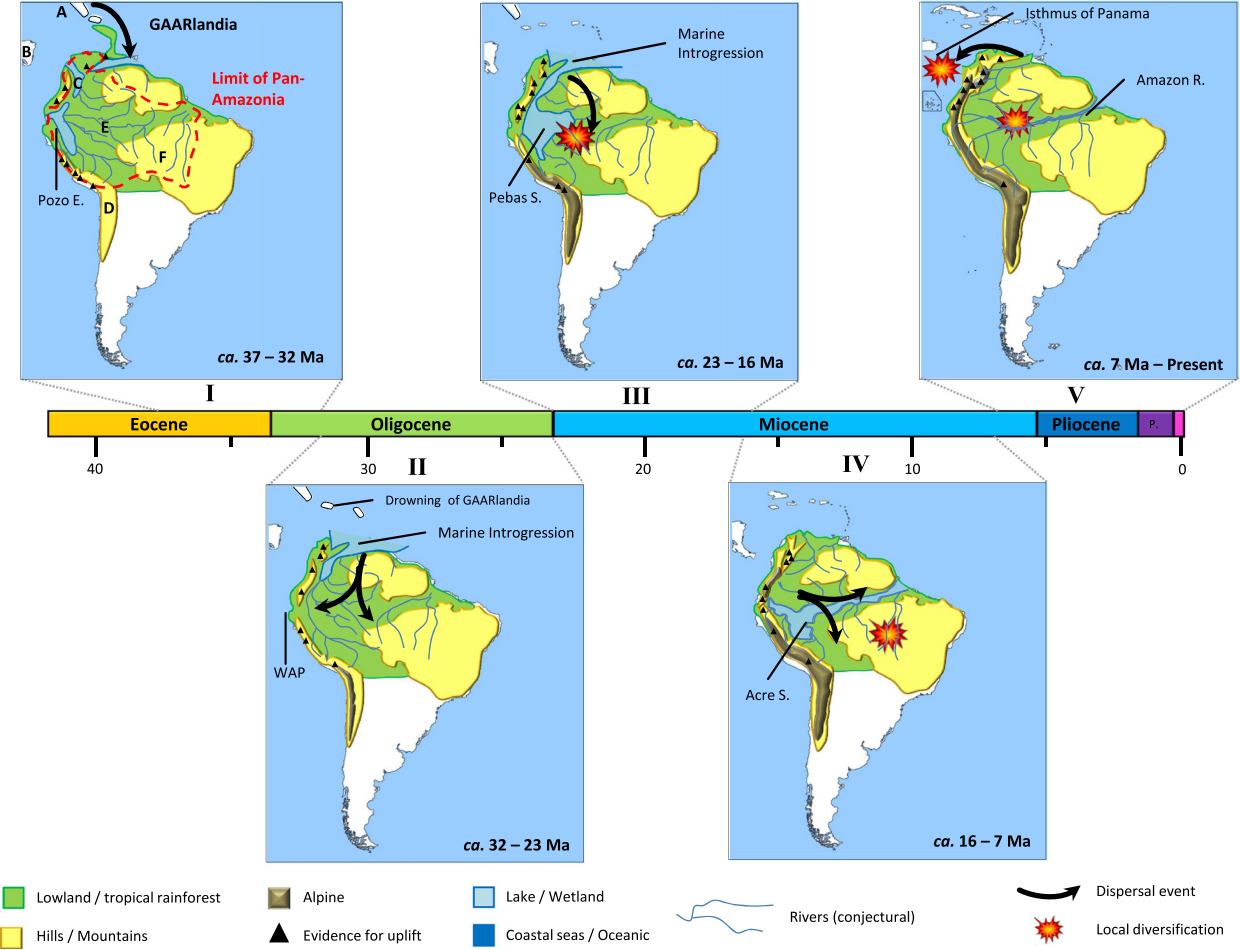
**Temporal and biogeographic evolution of Neotropical Troidini.** (I) Eocene-Oligocene boundary: Neotropical Troidini ancestors reached northern South America using intervening islands such as GAARlandia. (II) Oligocene: Amazonian and North Andean lineages become isolated by marine introgression such as the western Andean portal (WAP), thus leading the diversification near western Amazonia. (III) Early Miocene: Andean uplift created the Pebas System in western Amazonia, an extensive wetland that allowed steady diversification. (IV) Middle Miocene: the Pebas System drained and the Acre system initiated the formation of the Amazon River. The Pebas System delimited the Amazon basin into northern and southern parts, promoting the evolution of new lineages. (V) Pliocene-Present: the Amazon River became established and the Isthmus of Panama emerged, which facilitated land dispersal and promoted speciation. Letters (A-F) refer to geographic entities delimited for biogeographic analyses (see inset in Figure 1).

Our biogeographic analyses permit investigation of the nature of the colonization routes that have been used to reach South America. Two hypotheses can be postulated *a priori*: either a dispersal event through Central America, by crossing the oceanic barrier between Central and South America (Panama Strait) [60], or a dispersal event through the Caribbean Sea using intervening islands as stepping-stones (GAARlandia bridge) [66]. Interestingly, for both lineages the biogeographic results provide more support for the GAARlandia route as a stepping-stone pathway to colonizing South America. Central America was never recovered as the most likely ancestral area for the crown of each Neotropical lineage (more than 2-log likelihood difference with the optimal area). Instead the Amazon forest was consistently recovered (ln*L* = −90.12 for each clade), thus suggesting that both clades colonized this area from the Nearctic region through GAARlandia rather than via Central America. It is worth stressing that our biogeographic stratified model did not influence this result as we used similar rates of dispersal to account for possible dispersal events toward South America via the GAARlandia bridge or the Central America region. In addition, the temporal time frame of colonization of South America is congruent with formation of the GAARlandia bridge and the emergence of numerous intermediate islands that formed a pathway between the Nearctic and South America (Figures 3 and 4; [15,58,66]).

We speculate that early Troidini dispersed from North America to South America via the temporary connection of the GAARlandia bridge during Eocene–Oligocene times, which spanned 35–32 Ma [66]. This land connection may have also been important for the evolution of Phyciodina and Euptychiina butterflies (Nymphalidae) [67,68 respectively]. Our hypothesis implies that early colonizers of North America went extinct and current Troidini in North and Central America are the result of later colonizations from South America (see below; Figures 3 and 4).

After their arrival in South America, Amazonia played a significant role in the early evolutionary history of the Troidini, especially as a driver of diversification. Strikingly, the biogeographic analyses show that the Amazon forest region is the main geographic area of diversification for the Neotropical Troidini genera (Figure 3). This result is not unexpected because the Amazon forest covered most of northern South America until the Andes initiated orogenesis 20–23 Ma [15]. We thus hypothesize that swallowtails reached South America in the northern part where Amazonian rainforest was already present and diversified (Figure 4; [15]). Meanwhile, the Andes rose and the tropical rainforest was subsequently confined to central South America. It is interesting to note that we observed the same pattern in swallowtails that tracked Amazonia, a trend explained by tropical niche conservatism [22] as shown by the maintenance of the ancestral area in the Amazonian region (Figures 3 and 4). This role for Amazonia is likely to be reinforced with more extensive taxon sampling, since several of the missing species of *Battus* and *Parides* are restricted to the Amazon Basin (e.g., *B. madyes* and *B. laodamas*, and 10 out of 13 species of *Parides*) [27,29].

### Diversification pattern, biogeography and the role of Amazonia in the Neotropics

The diversification of Neotropical biodiversity has long been subject to debates and assumptions, especially regarding explanations for the megadiverse richness of Neotropical insects (e.g., [6,12,15,17]). Few studies have investigated the evolutionary history of these organisms (but see [13]). How this biodiversity evolved through time and the mechanisms involved in shaping it remain largely unknown [16], and the role of the Amazon rainforest remains elusive [69].

Pleistocene refuges were long held to be responsible for Amazonian diversity [12], but over time an older origin for this biodiversity was proposed as molecular phylogenetic studies accumulated and demonstrated that diversification mostly predated the Pleistocene (e.g., [15]). Our molecular dating results corroborate this trend, as Neotropical Troidini appeared around 27 and 21 Ma. The overall shape of the LTT plot (Figure 3) is fairly linear during this time, a pattern generally associated with a constant rate of diversification [42,63]. Both approaches, γ-statistics and the likelihood-based method, confirmed that diversification rates have not significantly varied through time. The γ-statistics returned an observed value of γ = −0.1573 at *p* = 0.437, indicating that rates remained constant according to the standard value of −1.645 [64]. Based on the MCCR-test, which takes into account the missing taxon sampling, the trend was confirmed with a critical value of γ = −1.78 and *p* = 0.659. The likelihood-based method also supports a constant rate model of diversification because ΔAIC_RC_ = AIC_RC_ – AIC_RV_ = −0.617, and favours the pure birth model over the Yule-3-rates model (see Additional file 4 Table S1 for details). A likelihood ratio test performed on the two previously best-selected models confirms that the constant rate model is significantly supported (Likelihood ratio = 8.582; *p* = 0.0034). To confirm these results, a relative cladogenesis test, as implemented in the *geiger* package [61], was used to identify lineages with unusually slow or rapid diversification rates for all slices through the tree (with the Bonferroni correction and 0.05 as the cut-off for significant *p*-value). The test highlights that no shift in diversification rates is evident for the Neotropical Troidini.

We repeated all analyses for only the *Euryades* + *Parides* clade (excluding the *Battus* clade), since the Neotropical Troidini are not monophyletic (see above; [36]). We obtained very similar results to the whole clade analysis for (*i*) γ-statistics: γ = −0.091 at *p* = 0.464 (MCCR-test: critical value of γ = −1.951 and *p* = 0.615); and (*ii*) the likelihood-based method: with the pure birth as the best constant rate model (AIC_RC_ = 46.437) and the Yule-3-rates as the best rate variable model (AIC_RV_ = 47.165) therefore favouring the constant rate model (ΔAIC_RC_ = −0.728; likelihood ratio = 7.272; *p* = 0.007). The relative cladogenesis test also confirms that no rate shift occurs in this clade.

Interestingly, equivalent taxon-level LTT plots within different animal and plant groups generally do not fit a constant rate scenario of diversification [14]. Our result for Neotropical Troidini is therefore unexpected because of the numerous environmental changes occurring in the Neotropical region that could influence the diversification of organisms during the Neogene [15,69]. Such a contrasted pattern could potentially be the result of several factors linked to their tropical ecology. One hypothesis advanced to explain the high levels of diversity in tropical ecosystems is the ‘museum’ model of diversity, whereby lineages accumulated steadily through time due to constant speciation rate or low extinction rate [10,11]. The hypothesis is supported by the fact that the diversification model that best approximates our data is the pure birth model, where the speciation rate is constant and the extinction rate is null. In fact, we expect that Amazonia played a key role in the observed pattern of diversification. The biogeographic reconstruction shows that swallowtails originated in the Amazon forest when they reached South America, and further followed the tropical rainforest when it was constrained by geological and climatic changes created by the Andean uplift (Figure 4), a trend referred to as tropical niche conservatism [22]. Tropical rainforest is often depicted as being ecologically stable through time, and has thus favored the preservation of comparatively ancient lineages resulting from adaptive radiation, facilitating the continued accumulation of species diversity [3,4]. For new migrants, Amazonia was thus an ecological opportunity that promoted their diversification, as well as a formidable reservoir of biodiversity that prevented any dramatic changes in diversification rates through time. This pattern is also confirmed by the lack of shifts in diversification rates during the evolution of Neotropical Troidini at the main climate or geological events, as determined using the likelihood-based method (see Additional file 4 Table S1).

In contrast, other studies that focused on the evolutionary history of Neotropical butterflies and their diversification patterns (e.g., [59,70,71]) recovered significant variation in diversification rates. They attributed these shifts in diversification rates to possible adaptive radiation linked to host plant shifts leading to ecological speciation processes and subsequent increases in diversification rates. Indeed, the regions spanning the upper Amazon and eastern Andes are geographic sources of colonization for several plant clades [5-8] and also constitute major diversity hotspots for plants in general [1]. These regions thus offer greater potential for ecological speciation driven by host plant adaptation, a speciation mechanism considered important in butterfly diversifications (e.g., [72]).

Troidini exclusively feed on *Aristolochia*[33], a genus of plants that comprises 350–430 species mainly distributed in tropical and subtropical regions worldwide [27,28,32]. The *Aristolochia* host plant niche provides substantial opportunity for the diversification of Troidini, especially as the Neotropical Troidini tend to be opportunistic in their host plant use [34]. However, host shifts within the genus *Aristolochia* have not necessarily led to ecological speciation or adaptive radiation, in contrast with other groups of phytophagous insects that experienced more drastic (and numerous) host shifts [35,36,72]. Fordyce [35] also showed that diversification of *Aristolochia* had no effect on diversification rates of Troidini. Together, this evidence is consistent with constant diversification rates and suggests that the Neotropical Troidini are able to colonize all areas containing *Aristolochia*[34]. Hence, this biotic factor may not be as important to Troidini diversification as in other butterfly groups [35,59,68,69].

### Amazonia as current motor for speciation

Amazonia contains the most species-rich biota on Earth [1,69] and is characterized by recent speciation events indicating that organisms experience an ongoing diversification [12]. The results of our analyses of DNA-based species delimitation support this trend as they recovered additional molecular entities that may or may not (depending on how conservatively we interpret them) be considered as new putative species (Figure 2). The LTT plot based on the ultrametric tree indicated a sudden increase in branching rate toward the present, corresponding to the switch from interspecific to intraspecific branching events (see the inset plot in Figure 2). To fit the position of this switch, the method of Pons *et al.*[41] was applied to the ultrametric tree (Figure 2). The GMYC model was preferred over the null model of uniform branching rates (*log*L = 322.35, compared to null model *log*L = 333.96; 2ΔL = 23.22; *χ*2 test, d.f. = 3, *p* < 0.0001). The model is consistent with the switch in the branching pattern occurring at −0.037 (i.e., T of the ML solution; root arbitrarily assigned to 1), leading to an estimate of 30 putative species (Figure 2). Overall, we recovered five molecular entities (corresponding to five recognized species) for the genus *Battus*, two molecular entities (corresponding to two recognized species) for the genus *Euryades*, and 23 molecular entities (corresponding to 21 recognized species) for the genus *Parides* (Figure 2; see Additional file 5 Figure S1 for the test with EF-1α phylogeny). The species delimitation analysis is in agreement with both morphological and geographical criteria e.g., [27-29,33,34,39]. The confidence interval for the threshold value included 23 to 35 molecular entities with likelihood scores ranging from −324.30 to 324.33 respectively (i.e., estimates falling within 2 log-likelihood units of the ML solution).

These analyses also clarify the taxonomic status of the *P. panthonus* complex (e.g., [39]). Based on our phylogenetic analyses and species-delimitations, *P. burchellanus* is conspecific to *P. panthonus* (including all subspecies), and should be treated as a junior synonym of *P. panthonus jaguarae*. Although *P. aglaope* has been tentatively placed as a distinct species, *P. aglaope* may be regarded as a subspecies of *P. panthonus*. However, our results stress the need for more comprehensive taxonomic studies [73]. In particular, several putative new species may be revealed within the widespread South American *P. neophilus* and the more localised *P. proneus* in the Atlantic Forest [29]. Strikingly, recent speciation events are reconstructed as being located in the Amazonian region. These results testify to the on-going process of diversification occurring in the Amazon rainforest. The emergence of the Isthmus of Panama is often postulated as a motor of recent speciation events and thus responsible for an increase in diversification rates [70]. Our study does not support this hypothesis because (*i*) dating analyses predate the emergence of the geologic structure (even taking into account the 95% HPD); (*ii*) no change in diversification rates was detected in the last seven million years, which corresponded to the onset of the Isthmus of Panama [58]; and (*iii*) no putative new species was shown in Central America by applying the species delimitation analyses.

### Methodological limitations

Conclusions about the temporal nature of diversification depend upon the quality of the underlying data [37,63-65,74,75]. Incomplete taxon sampling is a potentially serious problem of this kind that is difficult to address. Missing lineages can lead to inaccurate phylogenetic reconstructions, and anomalous branch lengths can in turn bias dating analyses (e.g., [75]). In our study, we first re-define the species boundaries before performing biogeographic and diversification analyses. Such an approach provides more consistent classification of taxa (e.g., splitting or pooling species) and inferences concerning diversification rates. When possible, we have taken into account missing lineages by using Monte Carlo simulations to circumscribe this effect [64]. But even with some missing taxa, taxon sampling can still provide uncertainty in the selection of the best-fit model of diversification [75].

Another methodological bias is the estimation of divergence times, which may introduce error into diversification analyses or distort conclusions (e.g., [63-65,72,73]). Poor choice of calibration and use of inappropriate statistical distribution can lead to illusory dating results (e.g., [37]). Although diversification analyses rely on branching times of the chronogram [63-65,74,75], few studies have taken dating uncertainties into account.

Such methodological limitations constrain the certainty of our interpretations and provide areas for future investigation [74,75]. As long as the fundamental hypothesis testing nature of these analyses are kept in mind, however, they remain our best window into understanding the rich, deep past of the stupendous biological diversity of our planet.

## Conclusions

Understanding the origin and evolution of Neotropical biodiversity is a fascinating challenge that relies on investigating intricate patterns to disentangle the processes involved in diversification mechanisms. Species-level molecularly dated phylogenies constitute powerful tools to unravel such patterns. Here, we show that Amazonia had a central role in the origin and evolution of Neotropical Troidini. In particular, the vast and stable ecological opportunity offered by this tropical rainforest is best explained by the hypothesis that Troidini swallowtails evolved under the museum model of diversity. This suggests that the Amazonian fauna has an older origin than supposed, which is in agreement with recent syntheses [15,67]. Comparative biogeographic and macroevolutionary analyses are required to confirm this trend on other swallowtails (*Papilio* subgenus *Heraclides*) or butterfly groups (e.g., [68,74,75]).

Amazonia is not only the evolutionary source of this diversity but continues to play an important role in late speciation events. Our results underline the need for further studies using dense taxon sampling, with most of the described subspecies each being represented by several individuals. Such a large survey requires collaboration and extensive fieldwork in remote tropical areas, including fine morphological examinations and dense molecular study. All together, these data illustrate the processes that have shaped extant Amazonian biodiversity and, on a broader scale, Neotropical species richness.

## Abbreviations

AIC, Akaike information criterion; BI, Bayesian inference; BIC, Bayesian information criterion; BV, Bootstrap values; DEC, Dispersal-extinction-cladogenesis; GMYC, General mixed Yule coalescent; ML, Maximum likelihood; PP, Posterior probabilities.

## Competing interests

The authors declare that they have no competing interests.

## Authors’ contributions

FLC designed the research. FLC retrieved material or data from GenBank, formerly published by KLSB and FAHS. FLC performed the analyses. FLC wrote the manuscript, with revisions by KLSB, GJK and FAHS. All authors read and approved the final manuscript.

## Authors’ information

**Fabien Condamine** was a PhD student at the Centre de Biologie pour la Gestion des Populations, and is now a post-doctoral fellow in the Centre Appliquées de Mathématiques with Hélène Morlon. He is interested in numerous aspects of historical biogeography, especially on global patterns in biodiversity such as latitudinal diversity gradients. He aims to decipher the main evolutionary and ecological processes that have shaped the present pattern of biodiversity. **Karina Silva-Brandão** is a post-doctoral fellow at Universidade de São Paulo (ESALQ/USP) in Brazil and has been working with molecular diversity and phylogenetic relationships at several taxonomic levels of wild and pest species of Lepidoptera. **Gael Kergoat** is a research scientist who has focussed on understanding the evolution of phytophagous insects and their host plants. Professor **Felix Sperling** is interested in processes of evolution ranging from the diversification of major insect lineages to the formation of species boundaries.

## Supplementary Material

Additional file 1 Dataset S1. MrBayes files (COI, COII and EF-1α separated and all genes combined into a single dataset) for phylogenetic analyses with all taxon sampling used for this study and GenBank accession numbers. Click here for file

Additional file 2 **Dataset S2. Paleogeographical model used in this study, with five time slices reflecting the probability of area connectivity through time.** (TXT 2 kb) Click here for file

Additional file 3 **Dataset S3. Dated phylogeny of Neotropical Troidini: maximum clade credibility tree with median ages from the Bayesian uncorrelated lognormal method (implemented in BEAST) using nucleotide sequence data from 3 loci.** (TXT 34 kb) Click here for file

Additional file 4 **Table S1. Results of analyses exploring diversification rates.** A: Results of net diversification rates (speciation minus extinction; [62]) for Neotropical Troidini species for three values of extinction rates (ϵ). On the right, best-fit extinction rates are estimated by maximum likelihood analyses. B: Results for various diversification models using ΔAIC_RC_ test statistic [42]. These tests fit a specified set of rate-constant (RC) and rate-variable (RV) variants of the birth-death model to branching times. C: Results of branching times analyses testing for temporal diversification rate during the major climate changes (EOGM, LOWE, MMCO, and PPG) using a Yule model and likelihood analyses [42] as described in Winkler *et al.*[26] and Condamine *et al.*[36]. (DOC 79 kb) Click here for file

Additional file 5 **Figure S1. Results of species delimitation analyses using only the EF-1α gene to reconstruct the phylogeny.** The GMYC model was not preferred over the null model of uniform branching rates (*log*L = 183.989, compared to null model *log*L = 183.646; 2ΔL = 0.687; *χ*2 test, d.f. = 3, *p* = 0.876). (PPT 105 kb) Click here for file
