# Supplementary material for: Biogeographic and diversification patterns of Neotropical Troidini butterflies (Papilionidae) support a museum model of diversity dynamics for Amazonia
Source: BMC Evol Biol. 2012 Jun 12;12:82. doi: 10.1186/1471-2148-12-82 (PMC3464124; doi:10.1186/1471-2148-12-82)
Supplement: Additional file 4 — Table S1. Results of analyses exploring diversification rates. A: Results of net diversification rates (speciation minus extinction; [62]) for Neotropical Troidini species for three values of extinction rates (ϵ). On the right, best-fit extinction rates are estimated by maximum likelihood analyses. B: Results for various diversification models using ΔAICRC test statistic [42]. These tests fit a specified set of rate-constant (RC) and rate-variable (RV) variants of the birth-death model to branching times. C: Results of branching times analyses testing for temporal diversification rate during the major climate changes (EOGM, LOWE, MMCO, and PPG) using a Yule model and likelihood analyses [42] as described in Winkler et al.[26] and Condamine et al.[36]. (DOC 79 kb) [file 1471-2148-12-82-S4.doc]

**Table S2.** Results from analyses of diversification rates for the whole Neotropical Troidini (*Battus*, *Euryades* and *Parides* included).

| **A)** |  |  |  |  |  |  |  | |  |  | |  | | |  | |  | | |  | | |  | |  | | |  | |  | |
| --- | --- | --- | --- | --- | --- | --- | --- | --- | --- | --- | --- | --- | --- | --- | --- | --- | --- | --- | --- | --- | --- | --- | --- | --- | --- | --- | --- | --- | --- | --- | --- |
| **Relative extinction** | **Assume full taxon sampling** | | | | | **Take into account missing taxa** | | | | |  | | **Estimates of extinction rates** | | | | | | | | | | | | |  | | | | | |
|  |  |  | |  | |  | |  | | |  | |  | | Data | | | |  | | | | | | | | | | | | |
| **ε = 0** |  | 0.0799 | |  | | **0.0871** | |  | | |  | | Likelihood | | **- 99.72** | | | |  | | | | | | | | | | | | |
| **ε = 0.5** |  | 0.0744 | |  | | **0.0798** | |  | | |  | | ε | | **0.1** | | | |  | | | | | | | | | | | | |
| **ε = 0.9** |  | 0.0491 | |  | | **0.0466** | |  | | |  | |  | |  | | | |  | | |  | | | | | | | | | |
|  |  |  |  |  |  |  |  | |  |  | |  | |  | |  | |  | | |  | | |  | | |  | |  | |  |
|  |  |  |  |  |  |  |  | |  |  | |  | |  | |  | |  | | |  | | |  | | |  | |  | |  |
| **B)** |  |  |  |  |  |  |  | |  |  | |  | |  | |  | |  | | |  | | |  | | |  | |  | |  |
| **Best CR model = Pure Birth (AIC = 55.093), best RV model = Yule-3-rate (AIC = 55.709) and ΔAIC_RC_ = - 0.617** | | | | | | | | | | | | | | | | | | | | | | | | | | |  | |  | |  |
| **Model** | **Parameters** | | **Type** | **Likelihood** | | **r1/lam0** | **r2** | | **a** | **X** | | **k** | | **st** | | **AIC** | | **st2** | | | **r3** | | | **r4/st3** | | | **ΔAIC** | |  | |  |
| **Pure Birth** | **r1** | | **RC** | **- 26.546** | | **0.083** | **NA** | | **NA** | **NA** | | **NA** | | **NA** | | **55.093** | | **NA** | | | **NA** | | | **NA** | | | **0** | |  | |  |
| Birth Death | r1, a | | RC | - 26.546 | | 0.083 | NA | | 0 | NA | | NA | | NA | | **57.093** | | NA | | | NA | | | NA | | | **2** | |  | |  |
| DDL | r1, k | | RV | - 26.428 | | 0.098 | NA | | NA | NA | | 104.22 | | NA | | **56.857** | | NA | | | NA | | | NA | | | **1.765** | |  | |  |
| DDX | r1, X | | RV | - 26.532 | | 0.092 | NA | | NA | 0.042 | | NA | | NA | | **57.064** | | NA | | | NA | | | NA | | | **1.971** | |  | |  |
| SPVAR | lam0, mu0, k | | RV | - 26.561 | | 0.085 | NA | | NA | NA | | 0.001 | | NA | | **59.121** | | NA | | | NA | | | NA | | | **4.028** | |  | |  |
| EXVAR | lam0, mu0, k | | RV | - 26.548 | | 0.085 | NA | | NA | NA | | 0.001 | | NA | | **59.097** | | NA | | | NA | | | NA | | | **4.004** | |  | |  |
| BOTHVAR | lam0, mu0, k, z | | RV | - 26.561 | | 0.085 | NA | | NA | NA | | 0.001 | | NA | | **61.122** | | NA | | | NA | | | NA | | | **6.029** | |  | |  |
| Yule-2-rate | r1, r2, st | | RV | - 25.524 | | 0.092 | 0.037 | | NA | NA | | NA | | 1.865 | | **57.048** | | NA | | | NA | | | NA | | | **1.955** | |  | |  |
| Yule-3-rate | r1, r2, r3, st1, st2 | | RV | - 22.855 | | 0.089 | 3.779 | | NA | NA | | NA | | 2.409 | | **55.709** | | 2.398 | | | 0.043 | | | NA | | | **0.617** | |  | |  |
| Yule-4-rate | r1, r2, r3, r4, st1, st2, st3 | | RV | - 22.255 | | 0.083 | 0.175 | | NA | NA | | NA | | 3.124 | | **58.511** | | 2.409 | | | 3.779 | | | 0.043/2.398 | | | **3.418** | |  | |  |
|  |  |  |  |  |  |  |  | |  |  | |  | |  | |  | |  | | |  | | |  | | |  | |  | |  |
| **C)** |  |  |  |  |  |  |  | |  |  | |  | |  | |  | |  | | |  | | |  | | |  | |  | |  |
|  |  | **EOGM (origin-34 vs. 34-0 Ma)** | | | | **LOWE (33-24 vs. 24-17 Ma)** | | | | | | **MMCO (24-17 vs. 17-8 Ma)** | | | | | | | | | **PPG (17-8 vs. 8-0 Ma)** | | | | | | | | | |  |
|  |  | Likelihood ratio | | *p*-value | | Likelihood ratio | | | *p*-value | | | Likelihood ratio | | | | *p*-value | | | | | Likelihood ratio | | | | | | *p*-value | | | |  |
| **Neotropical Troidini** | | **1.098** | | **0.295** | | **0.592** | | | **0.442** | | | **1.235** | | | | **0.266** | | | | | **0.068** | | | | | | **0.794** | | | |  |
|  |  |  |  |  |  |  |  | |  |  | |  | |  | |  | |  | | |  | | |  | | |  | |  | |  |
