# Supplementary figures and images for: Biogeographic and diversification patterns of Neotropical Troidini butterflies (Papilionidae) support a museum model of diversity dynamics for Amazonia
Source: BMC Evol Biol. 2012 Jun 12;12:82. doi: 10.1186/1471-2148-12-82 (PMC3464124; doi:10.1186/1471-2148-12-82)

## Slide 1
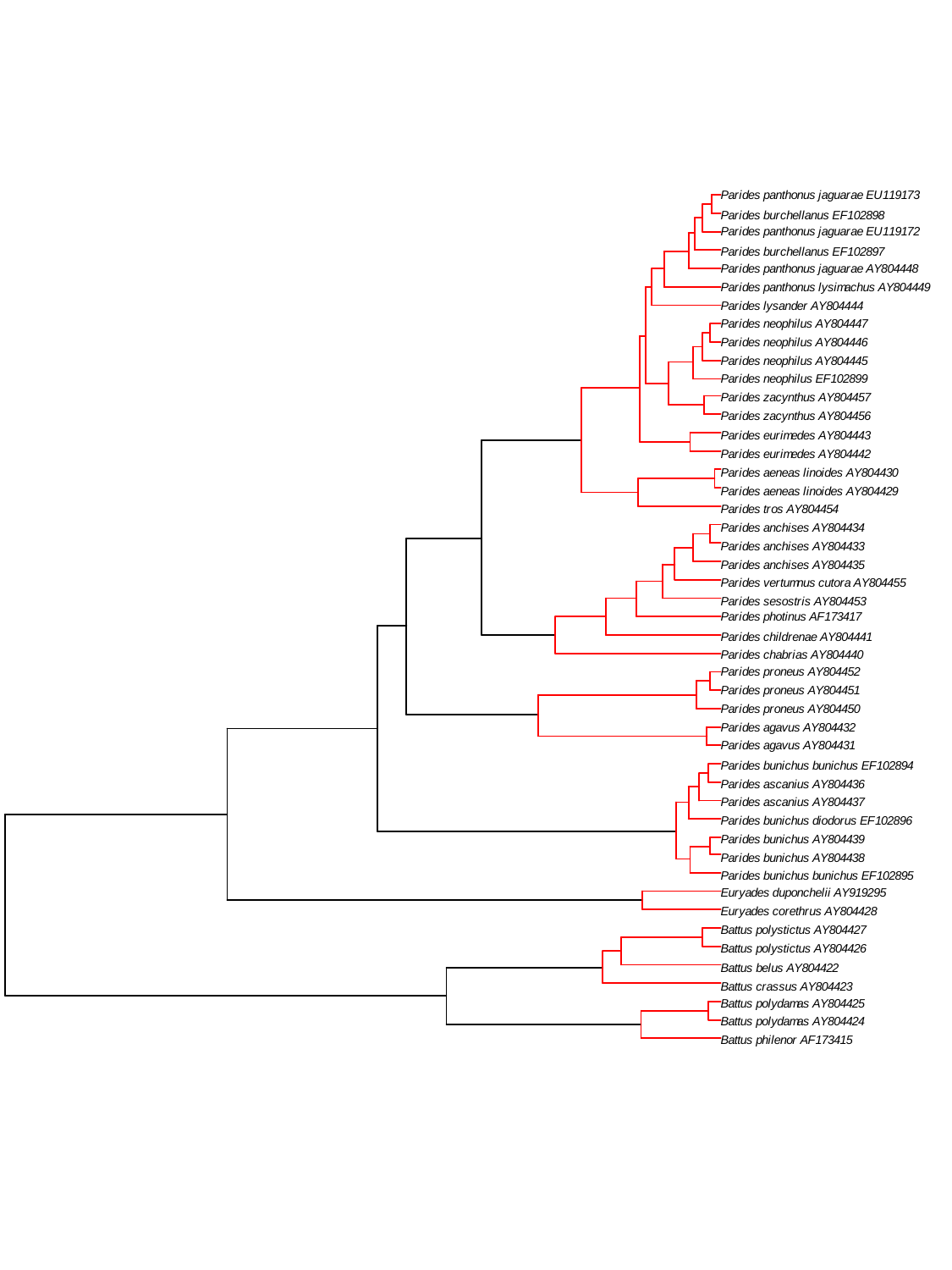

Supplement: Additional file 5 — Figure S1. Results of species delimitation analyses using only the EF-1α gene to reconstruct the phylogeny. The GMYC model was not preferred over the null model of uniform branching rates (logL = 183.989, compared to null model logL = 183.646; 2ΔL = 0.687; χ2 test, d.f. = 3, p = 0.876). (PPT 105 kb) [file 1471-2148-12-82-S5.ppt]
